# Nordic microalgae produce biostimulant for the germination of tomato and barley seeds

**DOI:** 10.1038/s41598-023-30707-8

**Published:** 2023-03-02

**Authors:** Teodor Alling, Christiane Funk, Francesco G. Gentili

**Affiliations:** 1 Department of Forest Biomaterials and Technology, Swedish University of Agricultural Sciences, 901 83 Umeå, Sweden; 2 Department of Chemistry, Umeå University, 901 87 Umeå, Sweden; 3 Present Address: Department of Biology, Lund University, 223 62 Lund, Sweden

**Keywords:** Biotechnology, Plant sciences

## Abstract

Microalgal biomass may have biostimulating effects on plants and seeds due to its phytohormonal content, and harnessing this biostimulating effect could contribute to sustainable agriculture. Two Nordic strains of freshwater microalgae species *Chlorella vulgaris* and *Scenedesmus obliquus* were each cultivated in a photobioreactor receiving untreated municipal wastewater. The algal biomass and the supernatant after algal cultivation were tested on tomato and barley seeds for biostimulating effects. Intact algal cells, broken cells, or harvest supernatant were applied to the seeds, and germination time, percentage and germination index were evaluated. Seeds treated with *C. vulgaris*, in particular intact cells or supernatant, had up to 25 percentage units higher germination percentage after 2 days and an overall significantly faster germination time (germinated on average between 0.5 and 1 day sooner) than seeds treated with *S. obliquus* or the control (water). The germination index was higher in *C. vulgaris* treatments than in the control for both tomato and barley, and this was observed for both broken and intact cells as well as supernatant. The Nordic strain of *C. vulgaris* cultivated in municipal wastewater thus shows potential for use as biostimulant in agriculture, adding novel economic and sustainability benefits.

## Introduction

Microalgae are a diverse group of unicellular and simple multicellular photosynthetic microorganisms that are present in all ecosystems on earth^1^. Their limited growth requirements and high adaptability^2^ as well as their advantage of being utilized simultaneously for multiple technologies (e.g., carbon mitigation, biofuel production, and bioremediation) has led to microalgae gaining popularity in various biotechnological applications^3–6^.

### Microalgae in wastewater treatment

In addition to proving useful in food and fuel production, microalgae have also long been known to be highly efficient in wastewater treatment^7–9^, offering an additional important economic use^7^. Wild strains of the green alga *Chlorella* have been shown to efficiently take up both nutrients and metals from wastewater in varying stages of treatment^10^, and nitrogen (ammonium) removal efficiency of mixed algae in photobioreactors can reach at least 60%^11^, with 90% total nitrogen removal also reported^12^. In open ponds with wastewater, mixed microalgae consortia have been shown to efficiently remove nitrogen and phosphorous^13^ and even pharmaceuticals^14–16^ and heavy metals such as lead^5, 17^.

### Microalgae as* biostimulant*

Increasing food demands and changing environmental conditions pressure agriculture to increase yields without further harming ecosystems in the process^18^. Agricultural practices therefore must be improved by using fertilisers and crop stimulants that are sustainably produced with little or no negative impacts on soil and ecosystem health. Biostimulants are compounds derived from organic material that increase germination, yield or growth in plants by mechanisms other than nutrition^19^. Microalgae are attracting the interest of agrochemical industries and farmers based on their promising properties for use in agricultural technology.

Hormones that actively stimulate germination, growth, or fruit set of higher plants, such as cytokinins and abscisic acids, have been detected in several species from the microalgal group Chlorophyta^20, 22^. There is also direct evidence for the biostimulating and biofertilising properties of Chlorophyte biomass: the extract of *Acutodesmus dimorphus* has been found to positively affect the growth of tomato seedlings^23^, the extract of *Scenedesmus obliquus* stimulates root growth and germination rate in common economical crops^24^, and the extract of *Chlorella vulgaris* raises seedling vigour in beet seeds^25^.

This study investigated the potential of two Chlorophyte microalgal strains as biostimulant for economically important food crops. Since microalgal strains vary in their biochemical properties, it is of value to test the biostimulating potential of different isolated strains. In this study, we investigated the potential of two local Chlorophyte isolates from the region around Umeå, Northern Sweden, to act as biostimulant for seed germination. These strains have previously been shown to efficiently treat wastewater and produce biomass^12^. In this study, municipal wastewater was used for cultivation, adding a novel investigation into combining wastewater treatment with the application of the harvested biomass as a biostimulant.

### Aims

The aim of the present study was to evaluate the potential of microalgal biomass grown in untreated municipal wastewater as a source of biostimulant and thereby to combine sustainable wastewater reclamation with sustainable biostimulant production. The biostimulating effects of intact cells, broken cells and harvest supernatant on seeds of the economically important crops tomato and barley were compared.

## Materials and method

### Microalgae cultivation

A 16 L photobioreactor (PBR) (Wheaton, UK) was filled with untreated municipal wastewater (MWW) collected at a local municipal wastewater treatment plant (Vakin, Umeå). The wastewater was filtered through paper tissue to remove large particles^26^.

Two microalgal strains isolated in northern Sweden, *Chlorella vulgaris* (13–1) and *Scenedesmus obliquus* (B2-2)^27^, were used to grow microalgal biomass, and one batch was cultivated per algal strain. The PBR with MWW was inoculated with approximately 125 mL of 14-day-old culture of either *C. vulgaris* (13–1) or *S. obliquus* (B2-2) grown in BBM medium^28^. The PBR was then kept in a 16:8 light:dark regime receiving 130 µmol m^−2^ s^−1^ photosynthetically active radiation on its surface using fluorescent lamps. The PBR was stirred at approximately 75 rpm and kept at room temperature for 8 days, at which point the culture was in the late exponential-early stationary growth phase.

### Culture harvest and biostimulant preparation

The biomass in the PBR was harvested by centrifugation at 4500 rpm and 15 °C for 9 min in 0.5 L centrifuge bottles using an Avanti J-26 XP centrifuge (Beckman Coulter, Inc., USA). Every other round, the algal pellets were collected in 50 mL sterile plastic tubes. The supernatant (SN) was collected in a separate vessel, filtered through a 0.2 µm filter, and kept refrigerated.

The cell pellets in 50 mL tubes were further concentrated by centrifugation at 4000 rpm for 10 min in an Eppendorf centrifuge 5804 (Eppendorf, Germany). The final pellets (referred to as intact cells, IC) were kept at − 20 °C until use.

Cells of both strains were individually disrupted to produce broken cells (BCs). A small aliquot of each freshly harvested cell mass was weighed, frozen with liquid nitrogen and transferred to 1.5 mL Eppendorf tubes. To make broken cells, this aliquot was then ground in an MM400 ball grinder (Retsch GmbH, Germany) with small (1 mm ⌀) steel beads at 20 Hz. Between the 20 cycles of 2 min, the tubes were cooled on ice. Sufficient grinding was confirmed by microscope observation using a Leica DMi1 inverted microscope (Leica Microsystems, Germany).

The biostimulating effect was thus tested on intact cells (IC), broken cells (BC) and supernatant (SN). The biostimulating effect of sterile filtered SN was tested at concentrations of 100%, 50% and 10% (diluted with ultrapure H_2_O (Milli-Q®, Merck KGaA, Darmstadt, Germany)). IC and BC were each suspended in ultrapure H_2_O to concentrations of 0.5, 0.3, and 0.1 g L^−1^ dry biomass (Supplementary Fig. S1). These nine treatments were prepared using both algal strains and compared to a control consisting of distilled water, resulting in 19 treatments for each plant species.

### Plant material

The crop species *Solanum lycopersicum* (tomato) and *Hordeum vulgare* (barley) were used for biostimulant experiments since both species are of high agricultural value not only in Europe but also worldwide^29, 30^.

Seeds of *Solanum lycopersicum* “TOTEM” F1 were purchased from Weibulls Horto, Sweden (retailer’s reported germination success of 92%), and *Hordeum vulgare* “Kannas eko Vårkorn” (Lantmännen SW Seed AB, Sweden) was a kind gift from Mrs Boel Sandström at the Department of Agricultural Research for Northern Sweden, SLU. All seeds were washed prior to usage and surface sterilized by submerging seeds in 10 mL sodium hypochlorite solution (5%) for 10 min before rinsing twice with ultrapure H_2_O^23^.

This study complies with local and national guidelines.

### Biostimulant experiment

Two milliliters of treatment solution (IC, BC or SN; or distilled water for control treatment) and 1 mL of distilled water were deposited on Whatman grade 181 seed testing paper (Cytiva, Germany) fitted in the lid of a 90 mm Petri dish. Twenty seeds were placed on filter paper, and the bottom half of the Petri dish was put in place, filling the function of a raised lid. This was repeated for both plant species, and five dishes were prepared for each treatment, resulting in 100 seeds per plant-treatment combination. The dishes were placed in ambient room light, far from but still reached by natural light, at room temperature. They were watered with distilled water according to demand each day (approximately 2 mL), and their positions were shuffled every other day. The day of germination was recorded for each seed. Germination for tomato was defined by the radicle emerging at least 2 mm. Since barley embryos have a coleorhiza, which must be ruptured for the radicle to emerge, germination for barley was defined as a visible radicle or a coleorhiza protruding 1 mm or more.

The filter papers with tomato seedlings were carefully transferred onto petri dish bottoms filled with water-saturated vermiculite on day 6. On day 7, all tomato dishes were placed under light tubes at 75–90 µmol m^−2^ s^−1^ under a 16:8 light:dark cycle and 26 °C at the warmest place. Barley seeds were placed under light tubes and in petri dishes filled with water saturated perlite:vermiculite mix at a ratio of 12:1 on day 3.

Monitoring, watering, and shuffling continued daily. The experiment was terminated after 14 days for tomato and after 5 days for barley, as determined when at least 93% of seeds in each treatment had germinated. At termination, the height of the two tallest seedlings in each dish was measured from filter paper to the apex meristem. A germination index (GI) was calculated for each treatment, as described by Kader^31^, as an accurate and encompassing measure of germination success. This GI was expressed relative to the control by dividing each group’s GI with the control GI.

### Statistical analysis

All statistical analyses and graphics were carried out using R statistical computing version 3.6.1. Mean values of the response variables were compared between the 19 treatments using one-way ANOVA if the residuals were normally distributed and variance was homogenous; otherwise, Kruskal–Wallis rank sum tests were used. Normality of residuals was tested using Shapiro-Wilks tests and QQ plots. Homogeneity of variances was tested using Levene’s test. If overall differences in means were found, post hoc testing (Tukey’s multiple comparison of means following ANOVA; or Conover's multiple comparisons following Kruskal Wallis rank sum tests) was employed to analyse pairwise differences. Two-tailed hypotheses were employed throughout, and the value α = 0.05 was used to identify significant differences in the mean of the response variables. For germination percentage, ANOVAs were run for each day, and this multiple testing was adjusted for by multiplying each p-value by the number of tests.

## Results

The biostimulating properties of two Nordic microalgal strains, *C. vulgaris* (13–1) and *S. obliquus* (B2-2), grown in municipal wastewater for 8 days were investigated. Three different concentrations each of intact cells (IC), broken cells (BC) or the supernatant (SN) after algal growth were applied to tomato and barley seeds as described in Section “Materials and methods”.

### Tomato biostimulant

**Figure 1:**
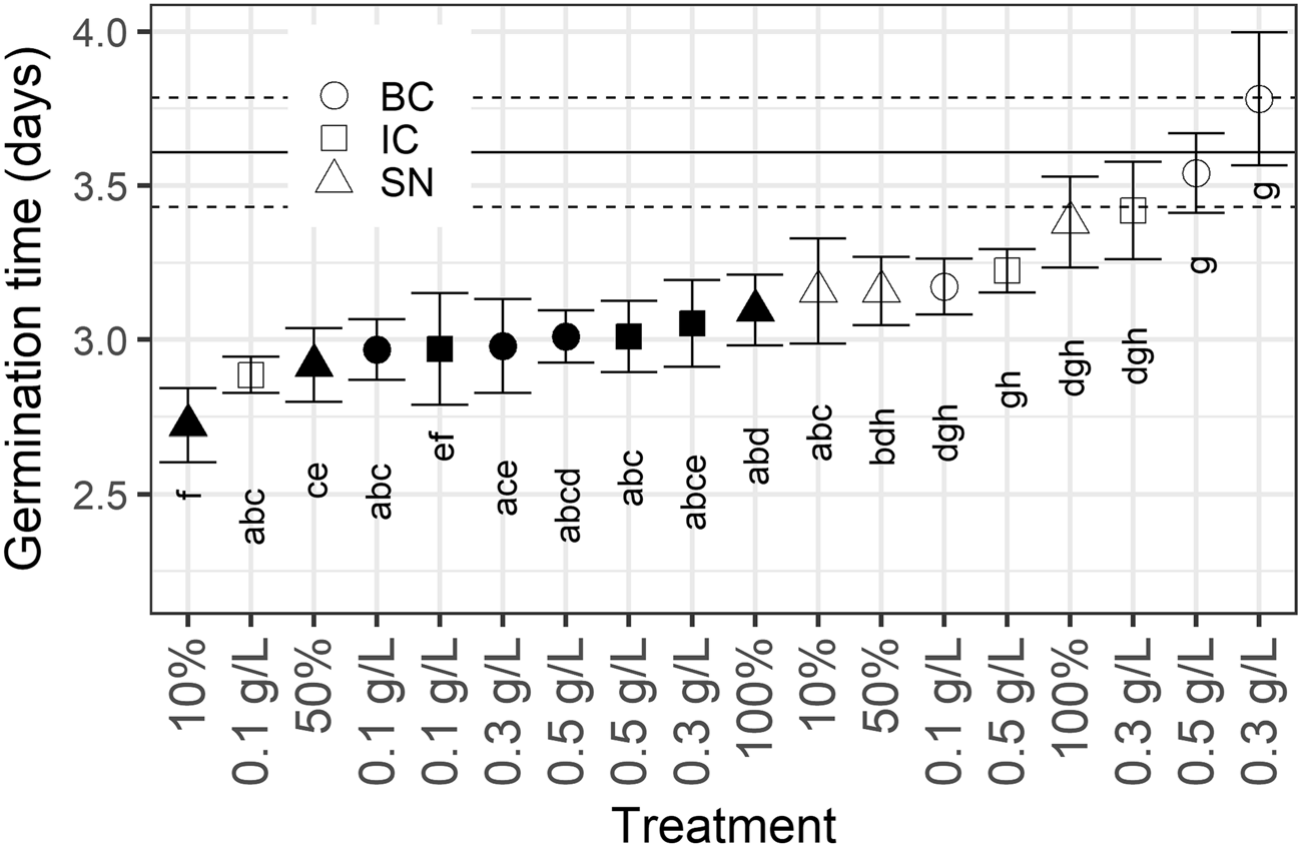
Germination times (in days) of tomato seeds. Shapes indicate mean of the 100 seeds for each treatment, filled shapes indicate *C. vulgaris* (13–1), open shapes indicate *S. obliquus* (B2–2). *IC* squares, *BC* circles, *SN* triangles. Letters below shapes indicate group belonging, within which no significant difference at the 0.05 level could be found (Kruskal–Wallis rank sum test with Conover’s post hoc test). Mean of the control group is indicated by the horizontal line, and its group adherence is “g”. Error bars indicate ± s.e.m. (in the case of control value indicator, ± s.e.m. is indicated by dashed horizontal lines).

Tomato seeds *(Solanum lycopersicum* “TOTEM” F1, Weibulls Horto, Sweden) were reported by the supplier to exhibit a germination rate of 92%. Indeed, they had very good viability with a high germination rate. There was an overall significant difference in mean germination time (Kruskal–Wallis rank sum test: χ_218_ = 179.3, p < 0.001) between treatments. All *C. vulgaris* (13–1) treatments resulted in a significantly faster germination time than the control (pure water) for tomato seeds, with at least 0.5 day faster germination (Fig. 1). Intact *S. obliquus* (B2-2) cells at 0.1 g L^−1^ and its supernatant at 10% and 50% concentrations also resulted in a faster mean germination time than the control. No other *S. obliquus* (B2–2) treatments were significantly faster than control (Fig. 1). All *C. vulgaris* (13–1)-treated groups had a higher GI than the control group, with broken cells at 0.3 g L^−1^ having the highest GI at approximately 9% greater than that of the control. *S. obliquus* (B2–2) broken cells at 0.1 and 0.3 g L^−1^ as well as intact cells at 0.3 g L^−1^ had an up to 2.5% lower GI than the control (Table 1).

**Table 1:** Germination index (GI) in tomato and barley seeds under the different treatments.

Tomato			Barley		
Strain	Treatment	GI	Strain	Treatment	GI
*Chlorella vulgaris* (13–1)	BC 0.3 g L^−1^	1.088	*Chlorella vulgaris* (13–1)	IC 0.1 g L^−1^	1.097
	IC 0.1 g L^−1^	1.067		IC 0.3 g L^−1^	1.069
	BC 0.5 g L^−1^	1.063		SN 10%	1.058
	IC 0.5 g L^−1^	1.063		IC 0.5 g L^−1^	1.056
	SN 50%	1.061		SN 100%	1.053
*Scenedesmus obliquus* (B2–2)	IC 0.1 g L^−1^	1.052		SN 50%	1.051
*Chlorella vulgaris* (13–1)	SN 10%	1.044	*Scenedesmus obliquus* (B2–2)	BC 0.1 g L^−1^	1.042
*Scenedesmus obliquus* (B2–2)	SN 100%	1.020		SN 10%	1.037
	SN 10%	1.018	*Chlorella vulgaris* (13–1)	BC 0.3 g L^−1^	1.025
	SN 50%	1.018		BC 0.1 g L^−1^	1.005
*Chlorella vulgaris* (13–1)	IC 0.3 g L^−1^	1.016	*Scenedesmus obliquus* (B2–2)	IC 0.1 g L^−1^	1.005
*Scenedesmus obliquus* (B2–2)	BC 0.5 g L^−1^	1.016	*Chlorella vulgaris* (13–1)	BC 0.5 g L^−1^	1.002
*Chlorella vulgaris* (13–1)	SN 100%	1.013	*Scenedesmus obliquus* (B2–2)	IC 0.5 g L^−1^	1.002
	BC 0.1 g L^−1^	1.002	n/a	Control (water)	1.000
*Scenedesmus obliquus* (B2–2)	IC 0.5 g L^−1^	1.002	*Scenedesmus obliquus* (B2–2)	BC 0.3 g L^−1^	0.991
n/a	Control (water)	1.000		IC 0.3 g L^−1^	0.991
*Scenedesmus obliquus* (B2–2)	BC 0.1 g L^−1^	0.995		SN 50%	0.991
	BC 0.3 g L^−1^	0.975		BC 0.5 g L^−1^	0.972
	IC 0.3 g L^−1^	0.975		SN 100%	0.963

GI values were calculated from the 100 seeds in each treatment and expressed relative to the value of the control group (water). Treatments are sorted within each seed type, from highest to lowest GI. Treatment name is given in the format ‘strain’ ‘cell type’ concentration’, where 13–1: *C. vulgaris*, B2-2: *S. obliquus*^27^ IC: intact cells; BC: broken cells; SN: supernatant. See Section “Materials and methods” for full details on how each treatment was produced.

**Figure 2:**
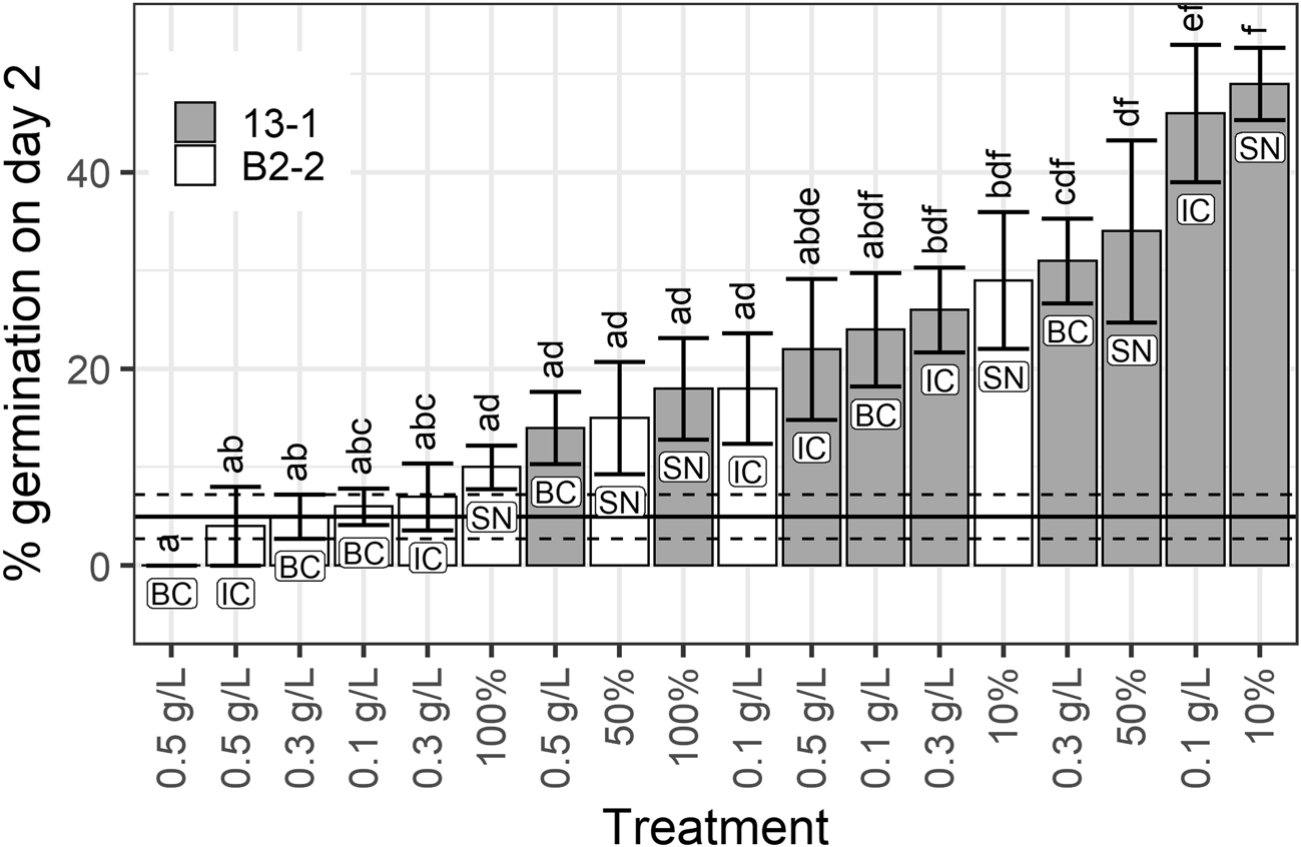
Percentage of tomato seeds germinated on day 2. Columns indicate the mean of the five dishes in each treatment, shaded columns indicate *C. vulgaris* (13–1), and white columns indicate *S. obliquus* (B2–2). Labels inside columns indicate the type of cell used for treatment. Letters above columns indicate group belonging, within which no significant difference at the 0.05 level could be found (one-way ANOVA with Tukey’s post hoc test). Mean of the control group is indicated by the horizontal filled line, and its group adherence is ‘ab’. Error bars indicate ± s.e.m. (control group ± s.e.m. is indicated by dashed horizontal lines).

Significant differences in germination percentage among treatments (one-way ANOVAs, adjusted for multiple testing) for tomato seeds were observed on day 2 (F_18_ = 8.102, p < 0.001) and day 3 (F_18_ = 2.683, p < 0.05). On day 2, *C. vulgaris* (13–1) intact cells at 0.1 g L^−1^, broken cells at 0.3 g L^−1^ and supernatant at 10% and 50% concentrations treatments resulted in significantly higher germination than the control group, as revealed by Tukey’s multiple comparisons of means (Fig. 2. The germination percentage at day 2 was 5% in the control compared to some *C. vulgaris* (13–1) treatments: 31% in broken cells at 0.3 g L^−1^, 34% in the supernatant 50% concentration, 46% in the intact cells at 0.1 g L^−1^ and 49% in supernatant at 10% concentration (Fig. 2). By termination on day 14, all treatments had reached equal or higher germination compared to the supplier’s reported germination success rate of 92% (data not shown).

**Figure 3:**
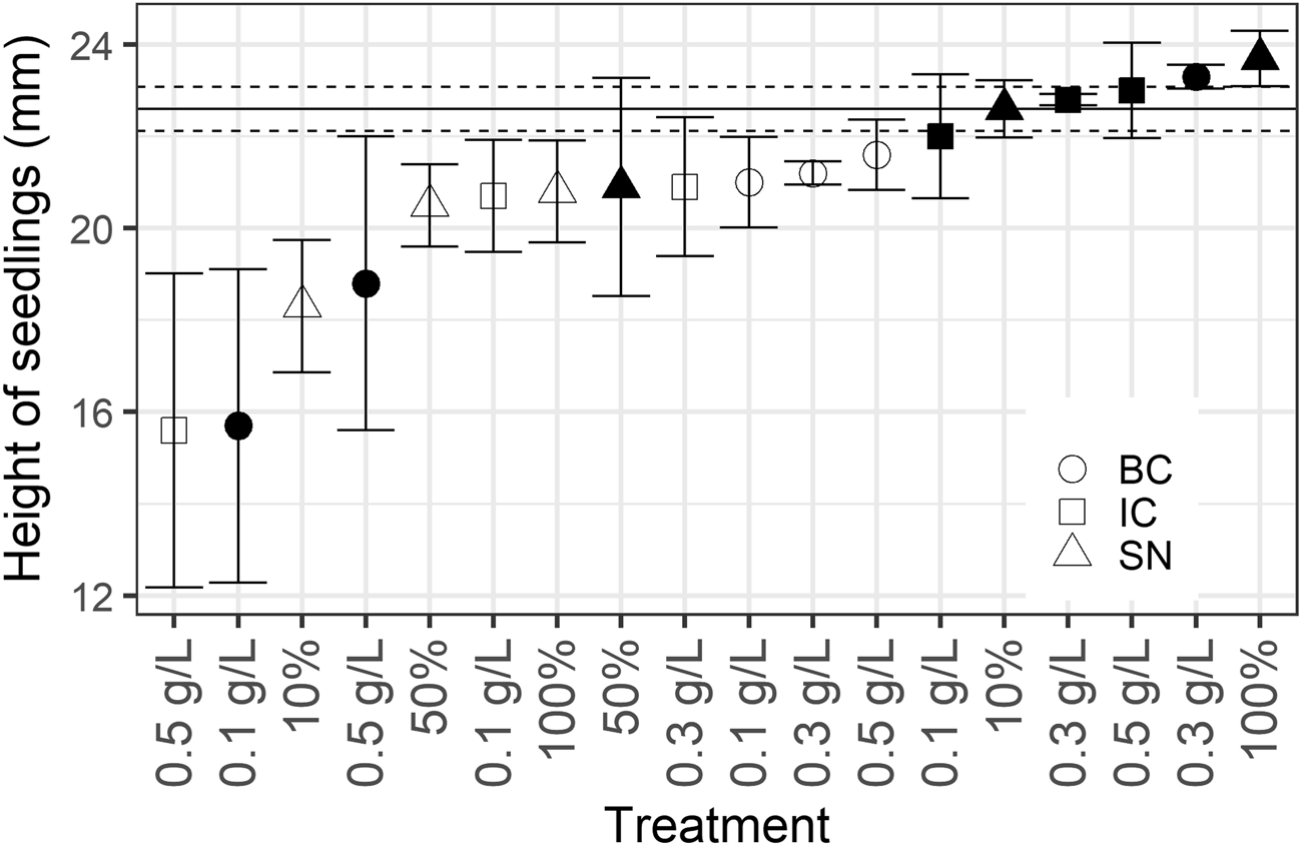
Height of tomato seedlings at day 14. Shapes indicate the mean of the ten measured seedlings from each treatment, filled shapes indicate *C. vulgaris* (13–1), open shapes indicate *S. obliquus* (B2–2). *IC* squares, *BC* circles, *SN* triangles. Mean of the control group is indicated by the horizontal line. Error bars indicate ± s.e.m. (control group ± s.e.m. is indicated by dashed horizontal lines).

The heights of the tomato seedlings also differed significantly at day 14 among the treatments (one-way ANOVA: F_18_ = 1.917, p < 0.03). Post hoc testing using Tukey’s multiple comparisons of means did not reveal any pairwise differences (Fig. 3).

### Barley biostimulant

**Figure 4:**
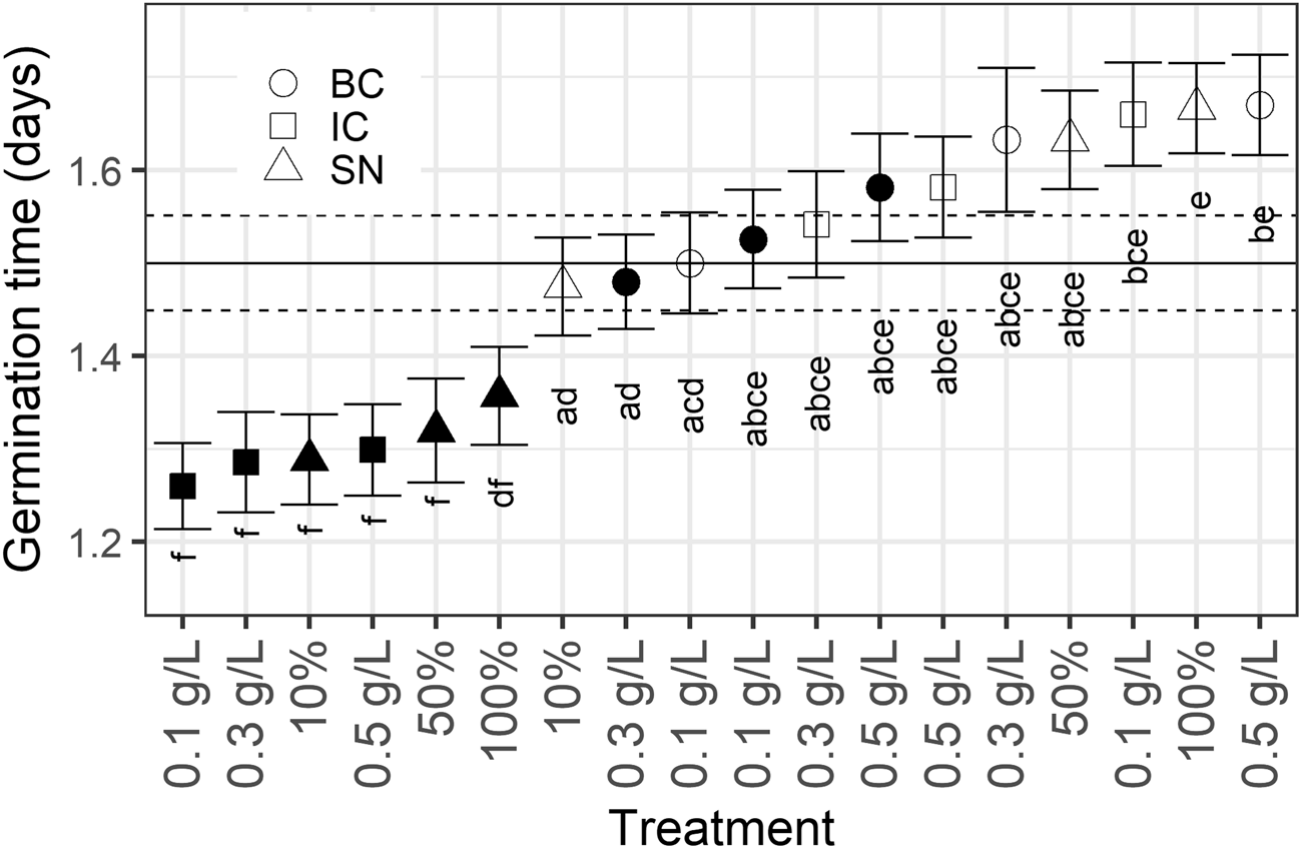
Germination times (in days) of barley seeds. Shapes indicate the mean of the 100 seeds for each treatment, filled shapes indicate *C. vulgaris* (13–1), open shapes indicate *S. obliquus* (B2–2). *IC* squares, *BC* circles, *SN* triangles. Letters below shapes indicate group belonging, within which no significant difference at the 0.05 level could be found (Kruskal–Wallis rank sum test with Conover’s post hoc test). Mean of the control group is indicated by the horizontal line, and its group adherence is “abcd”. Error bars indicate ± s.e.m. (in the case of the control value indicator, ± s.e.m. is indicated by dashed horizontal lines).

Barley seeds “Kannas eko Vårkorn” (Lantmännen SW Seed AB, Sweden) received the same treatments as previously described for tomato seeds. There was an overall significant difference in mean germination time between treatments (Kruskal–Wallis rank sum test: χ^2^_18_ = 133.12, p < 0.001). Post hoc testing using Conover's test for multiple comparisons of independent samples revealed a significantly faster germination time, up to about 0.25 days, for treatments with intact *C. vulgaris* (13–1) cells independent of the concentration. Additionally, the supernatant after *C. vulgaris* (13–1) harvest at 10% and 50% concentrations induced significantly faster germination, up to about 0.2 days compared to the control. Supernatant of *S. obliquus* (B2-2) at 100% concentration, on the other hand, resulted in a significantly longer mean germination time, up to about 0.16 days compared to the control treatment. No other treatments were significantly different from the control (pure water) (Fig. 4). Similar to tomato seeds, all barley groups under any *C. vulgaris* (13–1) treatment showed higher GI than the control, with intact cells at 0.1 g L^−1^ having almost 10% higher GI than the control. All treatments with lower GI than the control were *S. obliquus* (B2–2) treatments (Table 1).

**Figure 5:**
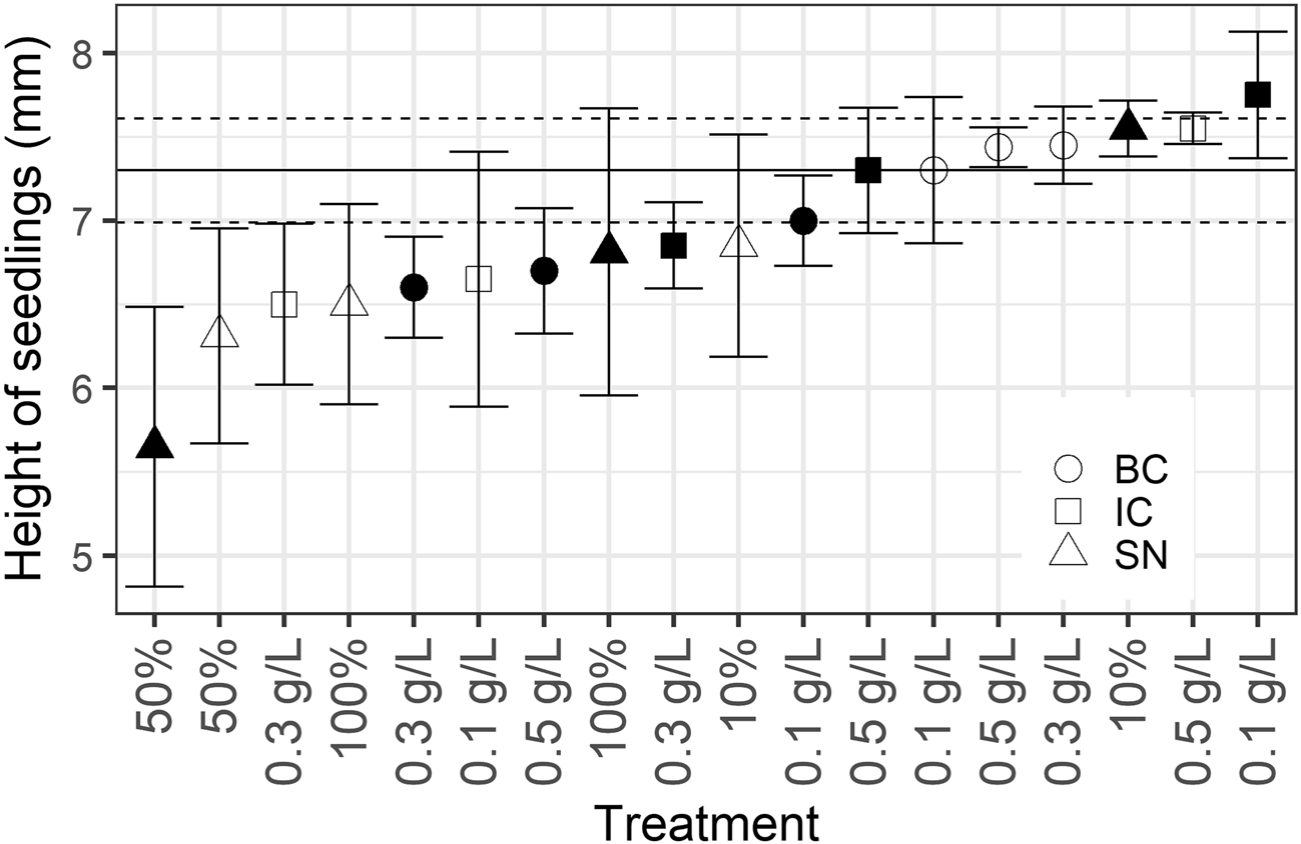
Height of barley seedlings at day 5. Shapes indicate mean of the ten measured seedlings from each treatment, filled shapes indicate *C. vulgaris* (13–1), open shapes indicate *S. obliquus* (B2–2). *IC* squares, *BC* circles, *SN* triangles. Mean of the control group is indicated by the horizontal line. Error bars indicate ± s.e.m. (control group ± s.e.m. is indicated by dashed horizontal lines).

At day 5, there was no overall significant difference in barley seedling mean height among treatments (one-way ANOVA: F_18_ = 1.225, p = 0.265) (Fig. 5).

## Discussion

In the present study, two different Nordic microalgal strains were grown in municipal wastewater. After harvesting, intact microalgae, broken cells or supernatant were applied to tomato and barley seeds, and germination time, germination percentage, germination index, and seedling height were evaluated. As will be discussed, it is reasonable to infer that biostimulating compounds were produced and excreted by the microalgae affecting seed germination and therefore provide a sustainable seed pretreatment product for use in agriculture.

The advantage of using microalgae as biostimulant has been documented on, e.g., watercress seeds, which displayed a greater germination index when treated with unhydrolyzed, undisrupted microalgal biomass compared to water^24^. In the present study, both tomato and barley seeds germinated faster and with higher GI under treatment with intact cells of the Nordic strain *C. vulgaris* (13–1) than the control, independent of the concentration. Interestingly, even the supernatant after algal growth of *C. vulgaris* (13–1) had a stimulating effect at 10% or 50% concentrations on the percentage germinated at day 2 for tomato seeds (Fig. 2) and at all concentrations on the overall tomato germination time (Fig. 1). In addition, all supernatant treatments yielded higher GI than the control (Table 1); for tomato, the supernatant from *C. vulgaris* (13–1) resulted in up to 6.1% higher GI than the control, and for barley, the GI was up to 5.8% higher than control. GI is a measure of germination success considering both germination speed and final percentage^31^, and it is interesting that supernatant has such a general positive effect on seed germination. The biostimulating effect of the Nordic *C. vulgaris* (13–1) on tomato seed germination at day 2 was almost 40% units higher for some treatments compared to control (Fig. 2). It should be noted that this significant effect was observed without any pretreatment of the biomass. We can exclude the possibility that germination-stimulating factors were already present in the wastewater before algal growth because the supernatant or cells of *S. obliquus* (B2–2) did not induce a similar effect as the supernatant or cells of *C. vulgaris* (13–1), even though both strains were cultivated in the same type of MWW.

The use of microalgal biomass to be used as a biostimulant for seeds germination has been recently shown also in other studies^32, 33^. However, the previous studies used extracted biomass of a south European strain of *C. vulgaris* to evaluate the stimulating effects on germination of watercress seeds^32, 33^. Hence, to the authors’ knowledge the use of supernatant after microalgal cultivation in untreated municipal wastewater has not been shown earlier. The use of MWW to grow microalgae for the production of biostimulant is important for future environmentally sustainable applications.

Several studies have been successful in using germination assays to test microalgal products as seed stimulants. The effluent of *Chlorella sorokiniana* cultivation did not have any obvious effect on the germination of lettuce seeds^34^. The *C. vulgaris* strain MACC-1 further could not stimulate the germination of watercress seeds when grown in centrate of municipal wastewater, but it could stimulate germination when grown in artificial medium BG-11^35^. While several studies showed the stimulation of seed germination using intact cells or extracts of microalgal biomass, the present study showed a significant stimulation of the germination of tomato and barley seeds solely using supernatant after microalgae cultivation in wastewater. Hence, using the supernatant allows recycling of the effluent after microalgae cultivation, leaving the entire biomass for other uses.

Biostimulating treatments with the Nordic *C. vulgaris* (13–1) strain were superior to treatments with the Nordic *S. obliquus* (B2–2) strain. These results concur with a previous study where cell extracts from a Dutch *C. vulgaris* strain had more stimulating effects than a Dutch *Scenedesmus quadricauda* strain on the germination of sugar beet^25^. Do et al.^22^ also reported up to two days shorter germination times for rice and tomato seeds pretreated with *Chlorella sorokiniana* extracts. Another study using a Portuguese strain of *S. obliquus* reported a ~ 39% higher germination index for watercress seeds treated with fresh biomass compared to a control^24^. It is possible that the brewery wastewater, in which those algae were cultivated, resulted in a different hormone/compound profile than the municipal wastewater used in our study. Alternatively, our Nordic *S. obliquus* (B2-2) strain might produce different phytohormones. Nonetheless, supernatant as well as fresh biomass from a close relative of *Scenedesmus*, *Acutodesmus dimorphus*, has also been shown to induce faster tomato seed germination^23^. However, BG-11 medium, not wastewater, was used to cultivate that algal biomass.

It is generally accepted that germination is caused by an increased ratio of gibberellic acid (GA) to abscisic acid (ABA) content in the seed^36^. A successful biostimulant (such as supernatant after *C. vulgaris* (13–1) cultivation) might therefore either (a) supply GA directly; (b) support GA biosynthesis; or (c) support ABA degradation. An increase in the germination index by microalgae has therefore been termed a “gibberellin-like” effect^24^. Eight different types of GA have been detected in three different *Chlorella* species, including *C. vulgaris*^21^. The *C. sorokiniana* strain used by^22^ was shown to produce significant levels of endogenous GA. Therefore, it is possible that cells, and even supernatant judging by its positive effect on germination, from our Nordic *C. vulgaris* (13–1) culture contain levels of GA high enough to elicit faster germination in both tomato and barely. Interestingly, a lower concentration (10%) of *C. vulgaris* 13–1 supernatant had a greater effect on germination time than undiluted (100%), but both treatments had significantly shortened germination time compared to the control (Fig. 1).

No overall difference in seedling height was observed between treatments, independent of the crop (Figs. 3, 5). However, a slightly greater height was measured for tomato seedlings treated with undiluted supernatant of *C. vulgaris* (13–1) (Fig. 3). Odgerel and Tserendulam^37^ applied *Chlorella* sp. biomass suspension to barley seedlings but did not observe any difference in seedling shoot height compared to control. Phytohormones should also stimulate postgermination, although it seems in this case that such an effect is negligible compared to the effect of the nutrients stored in each seed, which are used to establish roots and shoot postgermination. Germination-related response variables, as outlined in this study and others mentioned above, are useful when evaluating the effect of biostimulants on seeds. The potential postgermination growth-stimulating effects of microalgae should be further examined in future studies using more sophisticated assays.

## Conclusion

Cells and the supernatant after growth of the Nordic *Chlorella vulgaris* (13–1) strain in municipal wastewater were found to have a significantly stimulating effect on tomato and barley seed germination, while the effect of *Scenedesmus obliquus* (B2–2) was minor. The Nordic *C. vulgaris* (13–1) strain used might have some advantage over other *Chlorella* strains previously tested as biostimulant. Algal biomass and even supernatant therefore might be valuable products in agriculture as a contributor to sustainable food production. In future studies, it will be valuable to investigate the hormonal profile in different algal fractions, including the supernatant. Algal cultivation in municipal wastewater also offers a sustainable advantage over artificial media, and since similar cultivation systems under Nordic conditions have been proven to be efficient in wastewater purification^12, 13, 26, 27^, the results herein show promising sustainability and economic potential.

## Supplementary Information

Supplementary Figure S1.

## Data Availability

The datasets generated and analysed during the current study are available from the corresponding author on reasonable request.
