# Supplementary material for: Nordic microalgae produce biostimulant for the germination of tomato and barley seeds
Source: Sci Rep. 2023 Mar 2;13:3509. doi: 10.1038/s41598-023-30707-8 (PMC9981563; doi:10.1038/s41598-023-30707-8)
Supplement: Supplementary file 1 — Supplementary Figure S1. [file 41598_2023_30707_MOESM1_ESM.pdf]

Supplementary Information

Nordic microalgae produce biostimulant for the germination of tomato and barley seeds

Teodor Alling, Christiane Funk, Francesco G. Gentili

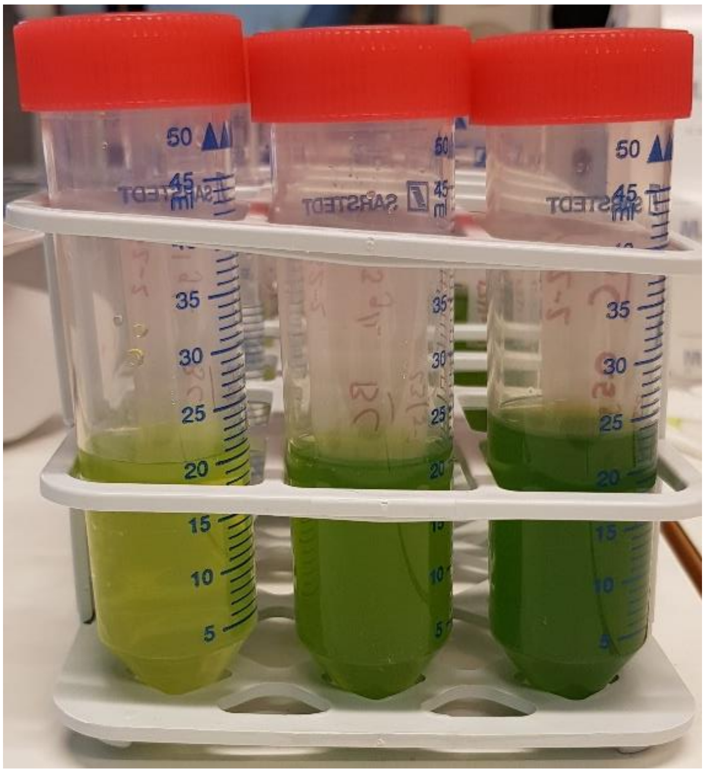

Supplementary Figure S1. The three concentrations of broken cell suspensions used in biostimulant experiments. From left to right: 0.1, 0.3, 0.5 g L^{-1} by dry weight.
